# A metabolomic study of the PPARδ agonist GW501516 for enhancing running endurance in Kunming mice

**DOI:** 10.1038/srep09884

**Published:** 2015-05-06

**Authors:** Wei Chen, Rong Gao, Xinni Xie, Zhibing Zheng, Haijing Li, Song Li, Fangting Dong, Lili Wang

**Affiliations:** 1 Beijing Institute of Pharmacology and Toxicology, Beijing 100850, China; 2 National Center of Biomedical Analysis, Beijing 100039, China

## Abstract

Exercise can increase peroxisome proliferator-activated receptor-δ (PPARδ) expression in skeletal muscle. PPARδ regulates muscle metabolism and reprograms muscle fibre types to enhance running endurance. This study utilized metabolomic profiling to examine the effects of GW501516, a PPARδ agonist, on running endurance in mice. While training alone increased the exhaustive running performance, GW501516 treatment enhanced running endurance and the proportion of succinate dehydrogenase (SDH)-positive muscle fibres in both trained and untrained mice. Furthermore, increased levels of intermediate metabolites and key enzymes in fatty acid oxidation pathways were observed following training and/or treatment. Training alone increased serum inositol, glucogenic amino acids, and branch chain amino acids. However, GW501516 increased serum galactose and β-hydroxybutyrate, independent of training. Additionally, GW501516 alone raised serum unsaturated fatty acid levels, especially polyunsaturated fatty acids, but levels increased even more when combined with training. These findings suggest that mechanisms behind enhanced running capacity are not identical for GW501516 and training. Training increases energy availability by promoting catabolism of proteins, and gluconeogenesis, whereas GW501516 enhances specific consumption of fatty acids and reducing glucose utilization.

Peroxisome proliferator-activated receptor (PPAR) are a class of ligand-dependent nuclear transcription factors that are important for metabolic homeostasis^1^. PPARδ is one of three PPAR isotypes and expresses in adipose tissue, liver, skin, brain, and skeletal muscle. Recent research demonstrated that PPARδ regulates many different biological activities such as lipid and lipoprotein metabolism, mitochondrial respiration, skeletal reprogramming, thermogenesis, inflammation, keratinocyte differentiation, and wound healing^2^,^3^,^4^,^5^,^6^,^7^. GW501516 is a synthetic PPARδ-specific agonist that has been crucial to elucidate the physiological and pathophysiological functions of PPARδ and generated novel strategies to treat metabolic diseases^2^,^4^,^8^,^9^. Recently, the relationship between PPARδ and exercise tolerance has garnered significant attention.

Exercise increases skeletal muscle PPARδ expression in humans and rodents^2^,^10^,^11^,^12^. Overexpression of a constitutively activated form of PPARδ in skeletal muscle enhanced running performance in sedentary mice and increased the percentage of slow-twitch fibres, which have a more oxidative phenotype^13^. Likewise, the selective PPARδ agonist GW501516 also induced similar effects^5^,^13^. Molecular analyses revealed that PPARδ is involved in exercise-induced reprogramming of muscle fibres and skeletal muscle metabolism by regulating expression of genes associated with contractile proteins, mitochondrial biogenesis, and lipid oxidation^14^,^15^. Decreased utilization of carbohydrates as energy and a proportional increase in fat oxidation were presumed to be the central mechanism for PPARδ-enhanced physical endurance and this was confirmed by observing a reduced respiratory exchange ratio in GW501516-treated animals^2^,^5^,^13^,^14^. However, it remains unclear how PPARδ activation drives metabolism toward fatty acids as the preferred energy source and which fatty acid pathway are affected by PPARδ activation.

Metabolomics (also known as metabonomics) has been recognized as a powerful tool to provide instantaneous snapshots of metabolic physiology by simultaneously analysing several metabolites^16^. Metabolomics has greatly extended our understanding of disease and systemic responses to therapeutic intervention. This study investigated the metabolomics associated with exhaustive running endurance following exercise training and/or GW501516 treatment in 4 groups of Kunming mice: untrained (sedentary) and untreated mice (NN group); untrained mice treated with GW501516 (NG); trained and untreated mice (TN); and trained mice treated with GW501516 (TG). Serum fatty acid metabolomic profiling and whole serum metabolomic profiling by two-dimensional gas chromatography time-of-flight mass spectrometry (GC × GC-TOFMS) and GC-TOFMS were used to investigate the metabolic phenotypes resulting from training, GW501516 treatment, and their combination. Results demonstrated that GW501516 administration for 3 weeks increased the running performance of both trained and sedentary KM mice. Furthermore, after exhaustive running, either training or GW501516 treatment promoted fatty acid oxidation and catabolism. However, GW501516-treated mice preferentially metabolized fatty acids, which resulted in reduced glucose consumption and lactate formation, while mice that were trained also utilized protein and glucogenic amino acids as energy sources.

## Results

### GW501516 increased the running distance of sedentary and trained KM mice on endurance tolerance tests

**Figure 1:**
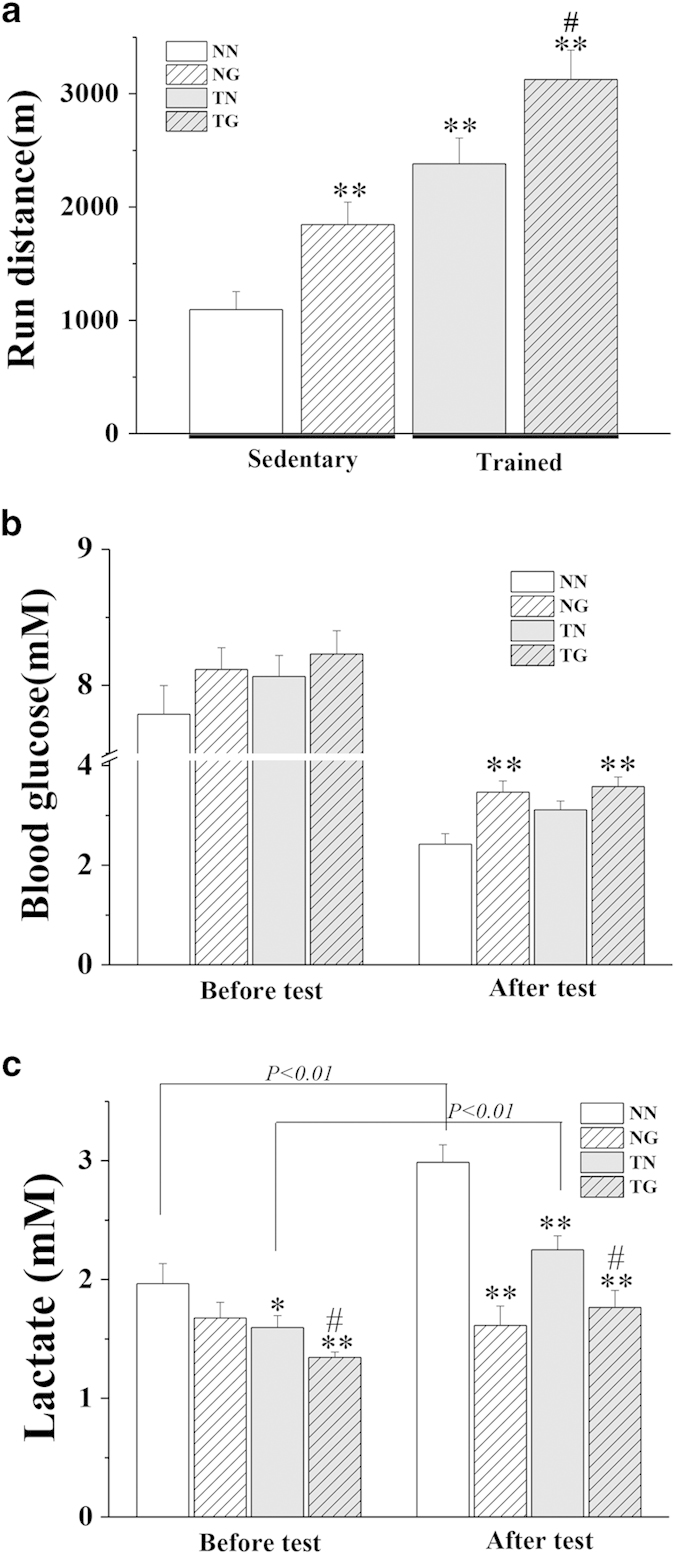
The effects of GW501516 on running performance, blood glucose, and blood lactate in sedentary and trained KM mice. Forced wheel running endurance tests were performed following 3 weeks of treatment. (**a**) shows the total distance ran by mice in each group. Blood glucose (**b**) and serum lactate (**c**) levels are shown for all groups both before and after running tests (n = 10–11 per group).*p < 0.05, **p < 0.01 compared to the NN group; #p < 0.05 compared to the TN group.

To examine whether GW501516 can enhance the running endurance capacity of mice, we treated age-matched wild-type KM cohorts with either vehicle or GW501516 for 3 weeks. Trained mice increased their exhaustive running distance by 744.4 m (31.2%) after 3 weeks of GW501516 treatment (TG versus TN) (Fig. 1a). GW501516 treatment for 3 weeks in sedentary KM mice also significantly enhanced running distance by 750.3 m (68.6%) compared to untrained control mice (p < 0.01 for NG versus NN). Similarly, even GW501516 treatment alone (NG group) for only 1 week improved the running distance by 671.8 m (48.6%) compared to the vehicle control group (NN group) (Supplementary Fig. S1a online).

### Effects of GW501516 on blood glucose and lactate levels in sedentary and trained mice

Before endurance tolerance tests, blood glucose levels in the trained and untrained GW501516-treated mice (TG or NG) and trained control mice (TN) tended to be higher than the untrained control mice (NN), but no statistically significant differences were observed (Fig. 1b). Blood lactate was slightly decreased in treated sedentary mice and untreated trained mice compared to untrained sedentary mice (p < 0.05 for NG or TN versus NN) (Fig. 1c). Training and GW501516 treatment further decreased blood lactate levels compared to untrained sedentary mice (p < 0.01 for TG versus NN). Exhaustive endurance test in sedentary untreated mice reduced blood glucose levels by 69% (p < 0.01) and increased blood lactate levels 52% (p < 0.01) compared to before testing. Exercise training alone for 3 weeks significantly reduced the elevation of blood lactate (0.65 mM increase in TN mice versus 1.02 mM increase in NN mice) after exhaustive running. Additionally, GW501516 treatment reduced utilization of blood glucose during endurance running (p < 0.01 for TG versus TN and NG versus NN). Blood lactate did not increase in untrained GW501516-treated mice (NG) after running (change of −0.06 mM) and was modestly, but significantly increased in trained GW501516-treated mice (TG) (change of 0.42 mM, p < 0.05) after running. Blood glucose levels of untrained GW501516-treated mice were higher than untrained controls (p < 0.01 for NG versus NN). Post-exercise blood lactate was markedly lower in trained GW501516-treated mice compared trained controls (p < 0.05 for TG versus TN) and lower in sedentary GW501516-treated mice compared to sedentary controls (p < 0.01 for NG versus NN). Furthermore, similar results were obtained after only 1 week of GW501516 administration (Supplementary Fig. S1 online).

### Serum fatty acid metabolomics

**Figure 2:**
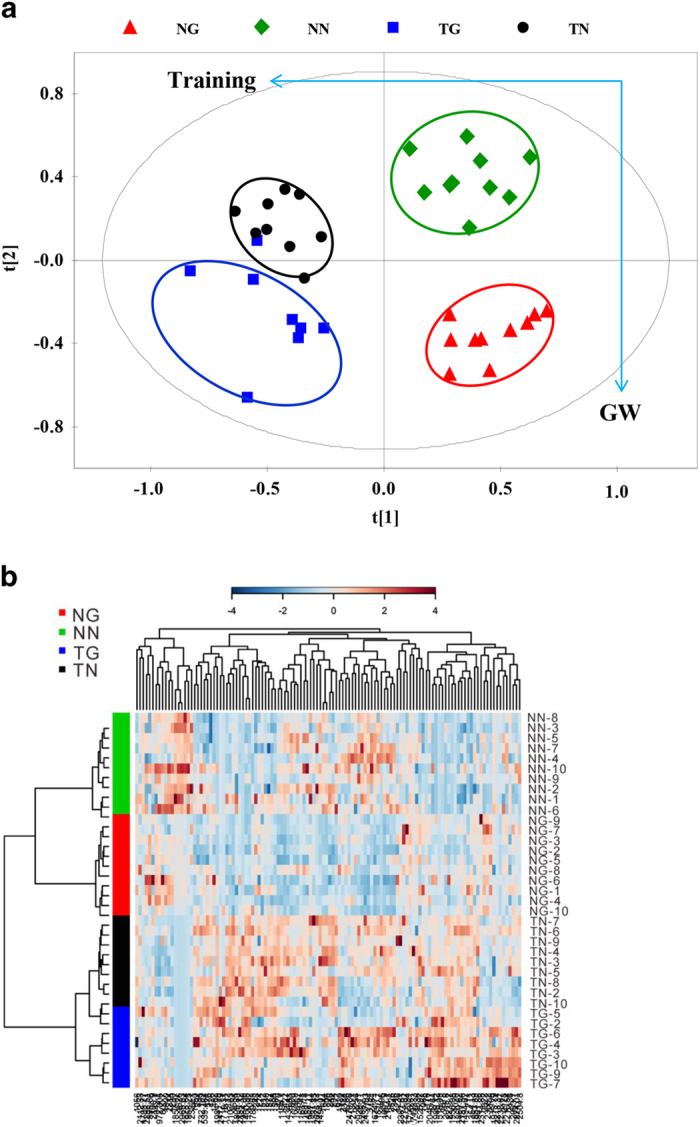
Metabolomic analysis of serum fatty acids. (**a**) shows PCA scores trajectory plots, (**b**) shows the representative heat map of untrained (NN and NG) and trained (TN and TG) mice. In the heat map, samples are sorted by row and metabolites are sorted by column. Green diamond, NN; Red triangle, NG; Black circle, TN; Blue square, TG;

**Figure 3:**
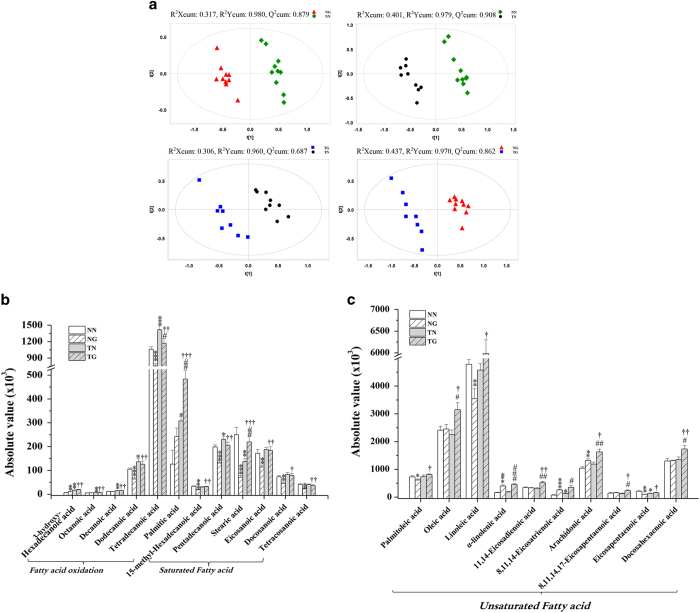
Differences in metabolite concentrations between groups. (**a**) shows PLS-DA score plots of groups based on serum spectral data from GC × GC–TOFMS. The score plots distinctly cluster the GW501516 treatment groups (NG and TG) compared to either control group (NN and NG). Furthermore, the trained groups (TN and TG) are distinctly clustered compared to their respective controls (NN and TG). (**b**) and (**c**) show changes in the relative concentrations of metabolites in the four groups. Two-tailed parametric *t* tests were used to compare concentration changes in each metabolite between groups (n = 10–11 per group). *p < 0.05, **p < 0.01 compared to the NN group, #p < 0.05, ##p < 0.01 versus TN; †p < 0.05, ††p < 0.01 compared to the NG group.

Fatty acid metabolomic analyses were performed on serum from exhausted mice by GC × GC–TOFMS. After data alignment, normalization and unit variance scaling, principle component analysis (PCA) score plots revealed a trend of intergroup separation and intragroup aggregation (Fig. 2). Results from a PLS-DA model by using two predictive components (R2X cum = 0.353, R2Y cum = 0.877, and Q2Y cum = 0.803 for sedentary groups; R2X cum = 0.306, R2Y cum = 0.960, Q2Y cum = 0.687 for trained groups) showed separations between GW501516-treated groups (TG and NG) and their respective control groups (TN and NN) (Fig. 3a). The metabolites with variable importance in the projection (VIP) values >1.5 and pvalues <0.05 were considered different between groups (Supplementary Table 1 online). These metabolites were subjected to computational systems analyses to investigate their roles in training and GW501516 treatment. The parallel PCA score trajectory plots (Fig. 2a), and heat map visualization (Fig. 2b) showed obvious differences resulting from training and/or GW501516. Relative to the distribution of metabolites in the untreated sedentary group (NN group) metabolites clustered along PC2 after GW501516 treatment alone (NG group) and above and to the right of the first component after training alone (TN group) (Fig. 2a). Combined GW501516 treatment and training generated a unique metabolic profile that that did not substantially overlap with the profiles of the individual treatments (training or GW501516 treatment alone). Scatter in the PCA plot for the trained and GW501516-treated group (TG) moved toward the training-only group (TN) along PC1, but the groups were distinct along PC2 (Fig. 2a).

**Figure 4:**
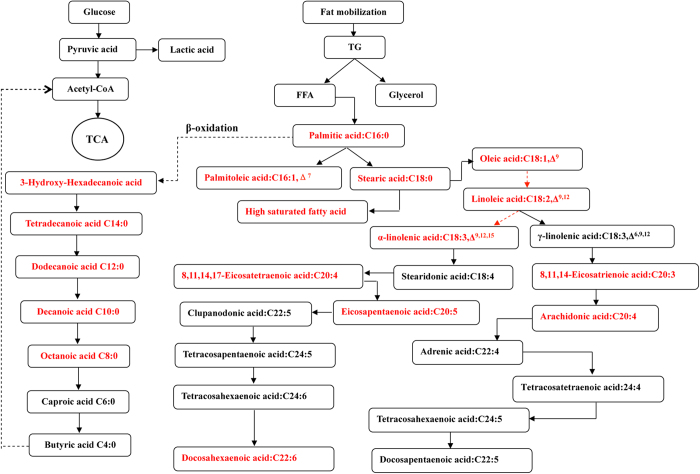
Pathway analysis of fatty acid metabolomics data Serum metabolites that were altered between groups are shown in red.

There were 10 types of fatty acids that differed between untreated trained mice and untreated sedentary mice (TN versus NN) (Fig. 3b,c, Supplementary Table 1 online). There were 15 fatty acids that were different between GW501516-treated sedentary mice and untreated sedentary mice (NG versus NN). There were 10 fatty acids that differed between trained mice receiving GW501516 treatment and untreated trained mice (TG versus TN). There were 20 fatty acids that were different in trained GW501516-treated mice compared to sedentary GW501516-treated mice (TG versus NG).Metabolites whose levels were different between groups could roughly be classified into 3 groups (constituents of fatty acid oxidation pathways, saturated fatty acid [SFA] and unsaturated fatty acid [UFA]). Palmitic acid, the predominate fatty acid *in vivo*, was increased substantially by training (143% greater in TN than NN) or GW501516 treatment (92% greater in NG than NN), and was further increased by their combination (282% greater in TG than NN) (Fig. 3b). Stearic acid was significantly decreased by training or GW501516 treatment alone, but which was reversed to level of NN group when training and GW501516 were combined (TG group), who had 60% more stearic acid than the trained untreated mice (TN group) and 208% more than untrained treated mice (NG group). The 5 kinds of fatty acids increased by training were all intermediates for β oxidation of long-chain fatty acids (Figs. 3b, and 4).Compared to sedentary controls (NN group), 6 out of 10 metabolites that were markedly reduced in the sedentary GW501516-treated group are long-chain saturated fatty acids (15-methyl-hexadecanoic acid, pentadecanoic acid, octadecanoic acid, eicosanoic acid, dodecanoic acid, and tetradecanoicacid) (Fig. 3, Supplementary Table 1 online). However, levels of the 6 saturated long-chain fatty acids reduced by GW501516 alone (NG compared to NN) were restored in trained mice that were treated with GW501516 (TG group) (Fig. 3b), suggesting higher rates of triglyceride hydrolysis after training and GW501516 treatment.

Another difference between training and GW501516 treatment is observed regarding UFA. GW501516 treatment alone markedly increased UFA and additional increases could be observed when combined with training (Fig. 3c). Relative to control mice (NN), only 1 polyunsaturated fatty acid (PUFA), 8,11,14-eicosatrienoic acid, was increased by training alone (TN) (79% greater in TN than NN), but 3 PUFAs, includingα-linolenic acid, 8,11,14-Eicosatrienoic acid and arachidonic acid, were increased by single GW501516 treatment (NG) (143%, 217% and 27% greater respectively in NG than NN). In mice that were trained, 7 of the metabolites (oleic acid, α-linolenic acid, 11,14-Eicosadienoic acid, 8,11,14-Eicosatrienoic acid, arachidonic acid, 8,11,14,17-eicosapentaenoic acid and docosahexaenoic acid) that were found to be different between GW501516-treated mice (TG) and trained controls (TN) were UFA and 6 of the UFA were PUFA (α-linolenic acid, 11,14-Eicosadienoic acid, 8,11,14-Eicosatrienoic acid, arachidonic acid, 8,11,14,17-eicosapentaenoic acid and docosahexaenoic acid). The above results indicated that exercise and GW501516 act synergistically to promote the mobilization of triglycerides and fatty acids oxidation.

### Whole serum metabolomics

**Figure 5:**
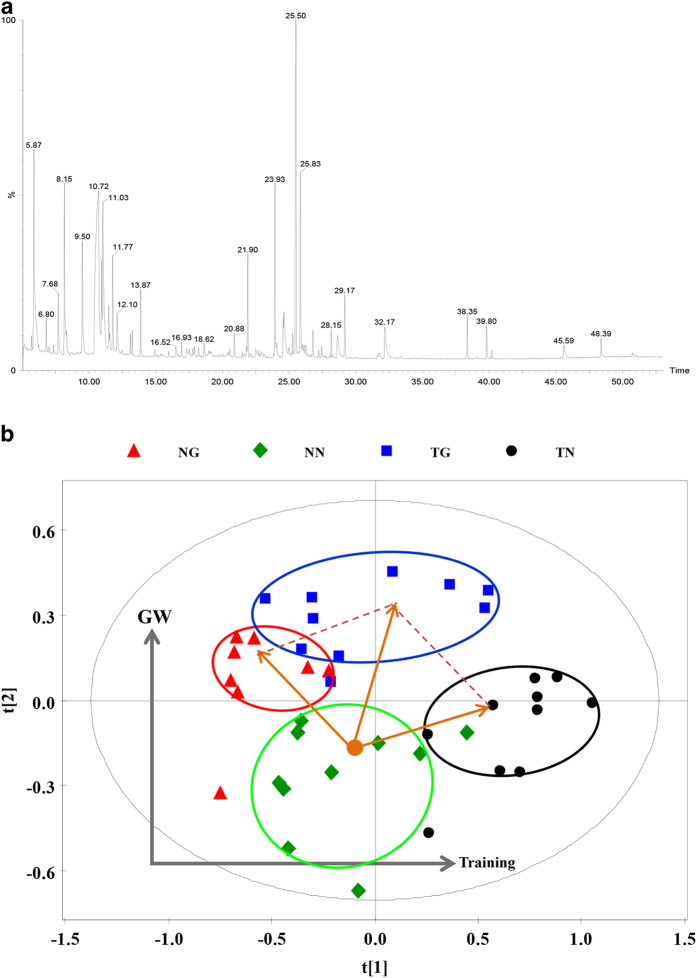
Changes in metabolites from whole serum. (**a**) shows the total ion chromatogram (TIC) of serum samples following GC-TOFMS. (**b**) shows the OPLS-DA score plot of serum samples for cross-validation.

**Figure 6:**
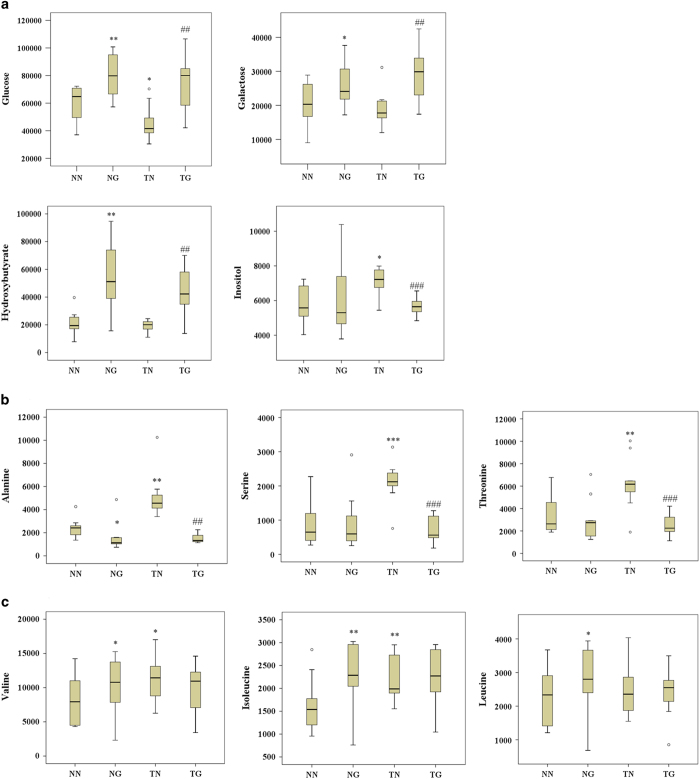
Metabolites that were significantly different between groups (**a**) shows serum glucose, galactose, hydroxybutyrate, and inositol. (**b**) shows serum glucogenic amino acid levels and (**c**) shows levels of branched chain amino acids (n = 10–11 per group). *p < 0.05, **p < 0.01 compared to the NN group; #p < 0.05 compared to the TN group.

To further define the metabolic response to GW501516 and/or exercise, a non-selective metabolomic study of serum was performed. A total of 122 peaks were obtained from the GC-TOFMS spectra (Fig. 5a), and 43 of those peaks were confirmed to be endogenous metabolites based on comparison with NIST02 libraries and commercially available reference compounds. The orthogonal partial least squares-discriminant analysis (OPLS-DA) cross-validated scores plots of whole metabolites in a way that was similar to those of the serum fatty acids metabolomics and showed clear segregations between groups (Fig. 5b). The distribution of the metabolites after GW501516 treatment (NG group) moved toward the second component relative to untrained sedentary mice (NN group). Training alone (TN group) caused metabolites to cluster to the right of those in the NN group, while training and GW501516 treatment (TG group) were directly above the NN group on the plot (Fig. 5b). Among the 43 metabolites, 14 were significantly different between groups and are involved in several key metabolic pathways such as fatty acid metabolism, galactose metabolism, glycogenic amino acid (alanine, serine and threonine) metabolism, branched chain amino acid (BCAA) (valine, leucine and isoleucine) metabolism, and metabolism of ketone bodies (Fig. 6). Changes in serum glucose and palmitic acid were in agreement with the other analyses performed in this study (Fig. 6a). Serum galactose and hydroxybutyrate were increased by GW501516 treatment regardless of training (TG and NG). Inositol however, was increased only after training alone (TN). Alanine, serine, threonine, valine, and isoleucine were increased by training, but leucine was not (Fig. 6a, and c). Moreover, the elevated levels of inositol and glycogenic amino acids seen with training alone (TN)were reversed by combining GW501516 treatment with training (TG). Additionally, BCAA were found to be consistently and significantly elevated by GW501516 treatment (Fig. 6c).

### Gene expression analysis

**Figure 7:**
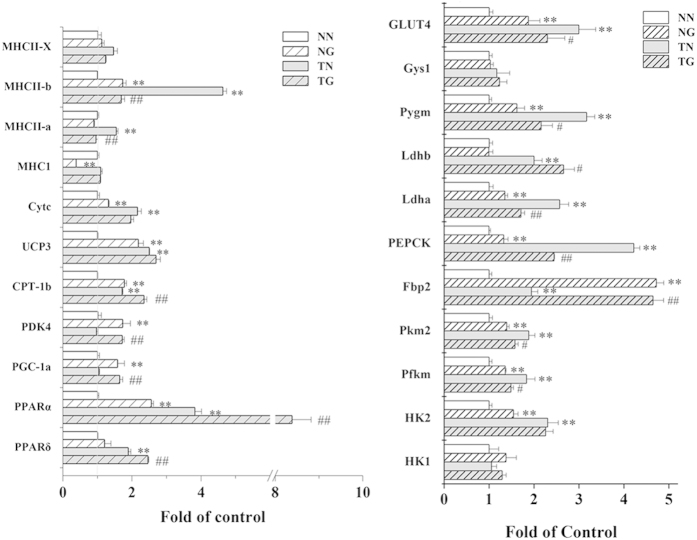
The effects of GW501516 treatment on skeletal muscle gene expression in trained and sedentary KM mice Gene expression was measured by QPCR (n = 4 per group). *p < 0.05, **p < 0.01 compared to the NN group; #p < 0.05, ##p < 0.01 compared to the TN group.

mRNA expression levels of PPARδ and PPARα were analysed by quantitative polymerase chain reaction (qPCR) (Fig. 7). qPCR analysis revealed that GW501516 treatment alone or in combination with training upregulated expression of PPARγ coactivator 1α (PGC1α) and pyruvate dehydrogenase kinase 4 (PDK4). Corresponding with this, more robust upregulating to genes involved in glucose supplementation (phosphoenolpyruvate carboxykinase1 (PEPCK1), fructose bisphosphatase 2 (Fbp2), and glycogen phosphorylase (Pygm)), glucose utilization and glycolysis (glucose transporters (GLUT4), hexokinase2 (HK2), phosphofructokinase (Pfkm), pyruvate kinase 2 (Pkm2) and lactate dehydrogenase (Ldh)) were found in untreated trained mice. Training and GW501516 together upregulated PPARα, muscle carnitine palmitoyltransferase Ib (CPT Ib), uncoupling protein 3 (UCP3) and cytochrome c (CYT-C). Upregulated PPARδ expression was observed only in trained mice (TG and TN), but greater expression was observed in trained mice that were treated with GW501516 than untreated trained mice. Similar expression patterns were also observed for PPARα and CPT Ib. Additionally, expression of the major histocompatibility complex I (MHC I) was down-regulated after GW501516 treatment alone (NG). Expression levels of MHCIIa and IIb were increased after training alone (TN), but these changes were reversed when training was combined with GW501516 treatment (TG).

### GW501516 increases the quantity of succinate dehydrogenase (SDH)-positive muscle fibres in both sedentary and trained mice

**Figure 8:**
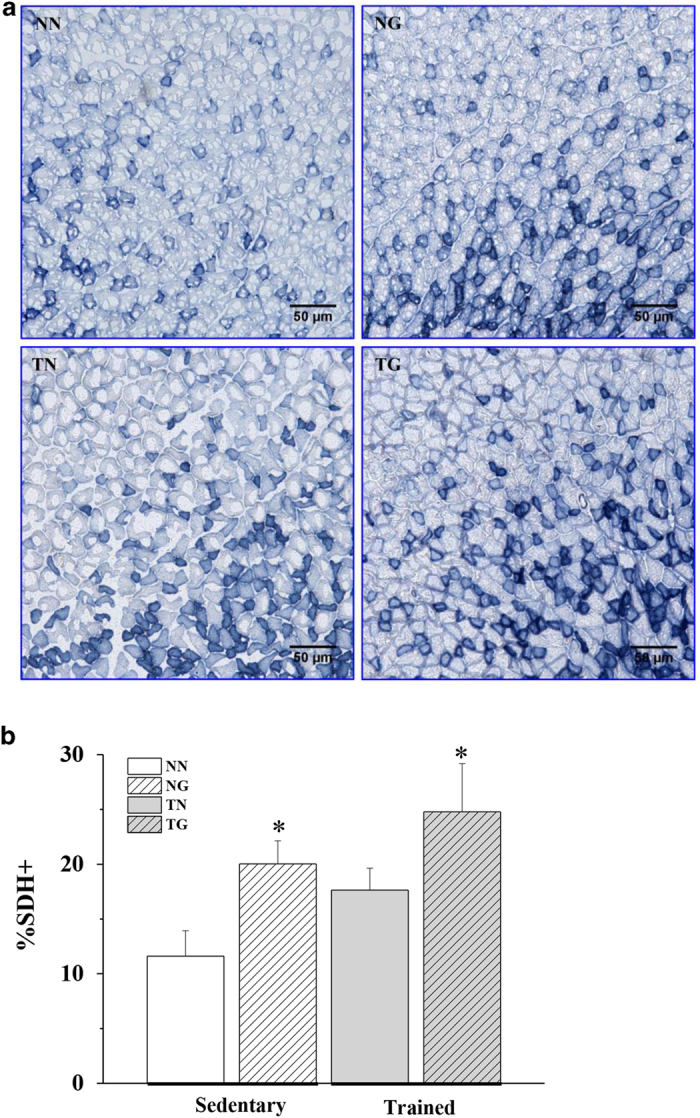
The effects of GW501516 on SDH-positive fibres in skeletal muscle of sedentary and trained KM mice (**a**) shows representative cross sections of a metachromatically stained gastrocnemius from each group of mice. Type-I fibres are stained blue. (**b**) shows quantification of SDH-positive fibres in all groups (n = 3). *p < 0.05 compared to the NN group.

SDH staining of gastrocnemius muscle sections revealed that there was a statistically insignificant trend for the proportion of SDH-positive fibres to increase following training alone compared to sedentary mice (TN versus NN) (Fig. 8). In contrast, GW501516 alone significantly increased the proportion of SDH-positive fibres by 72% compared to untreated sedentary mice (NG versus NN). Trained mice that were treated with GW501516 had 113% more SDH-positive fibres than untrained sedentary mice (TG versus NN) (Fig. 8b). Moreover the increase in SDH-positive fibres in trained mice treated with GW501516 occurred in a time dependent manner. Compared to the NN group, TG mice had 36% more fibres after 1 week and 82% after 2 weeks (Supplementary Fig. S2 online).

## Discussion

This study demonstrated that GW501516 increases exhaustive running performance and the proportion of SDH-positive muscle fibres in both trained and untrained mice. Slow-twitch myofibres are rich in mitochondria and have a high oxidative capacity. Formation of slow-twitch muscle fibres revolves around PGC-1α and its transcripts^17^,^18^. Like exercise, GW501516 alone is sufficient to improve running endurance of untrained mice, even after only 1 week of administration (Supplementary Fig. S1 online). Enhanced running endurance is the results of increased fatty acid utilization after PPARδ activation that upregulates PDK4 and other key components of fatty acid oxidation pathways. Accordingly, blood glucose levels of GW501516-treated mice were significantly higher after exercise while blood lactate was lower compared to untrained controls. Although GW501516 or exercise each enhanced fatty acid oxidation in skeletal muscle, GW501516 predominately induces fatty acid metabolism, while training also induced protein metabolism as an energy source.

Studies have demonstrated that GW501516 significantly increased the running performance of trained C57Bl/6J mice^5^, but not in untrained mice that were treated with 5 mg/kg over periods lasting 4 weeks to 5 months, or 3–30 mg/kg/day gavaged for 2 months^5^. Our study found that GW501516 increased running performance in KM mice regardless of training and the magnitude of improvement was unexpectedly greater for untrained mice. This finding could be due to different GW501516 administration protocols, exhaustive running protocols, or strain differences. In this study, running performance was determined by wheel running rather than treadmill running because it is more natural for mice, which are specialized climbers, and better measures their natural running abilities. Additionally, studies have shown significant differences in aerobic capacity, treadmill performance, and exercise training responses between different inbred and hybrid rodent strains^19^,^20^. The running distance of untreated trained mice (TN) was relatively higher than that of GW501516-treated untrained mice (NG). The effects of wheel training and the skills acquired during the training period cannot be ignored, but are difficult to control for.

PPARδ is the principle isoform involved in regulating muscle fuel utilization. Previous studies demonstrated that changes in expression of genes regulated by PPARδ occurred after 3 days of GW501516 treatment^5^. We also found that expression of PGC1α, PDK4, and selective biomarkers for fatty acid β oxidation in skeletal muscle were significantly increased by GW501516 treatment. Both palmitic acid and the earlier intermediate in the fatty acid oxidation pathway, 3-hydroxy-hexadecanoic acid, were elevated compared to untrained control mice. However, why the downstream products of fatty acid oxidation were not found to be increased in the GW501516-treated group warrants further investigation. Additionally, GW17046 (another PPARδ agonist) treatment in ob/ob mice markedly reduced levels of saturated long chain fatty acids (15–24 carbon atoms) in untrained GW501516-treated (NG) mice compared to untreated untrained (NN) mice^21^. This result suggests that unlike exercise, GW501516 is limited to promoting hydrolysis of triglycerides and that the increased serum palmitic acid observed in GW501516-treated (NG) mice may be due to the catabolism of saturated fatty acids. In contrast, the decrease in serum saturated fatty acids was completely reversed when GW501516 treatment was combined with training. Presumably, this is because of enhanced fat metabolism. GW501516 also increased blood glucose and lowered blood lactate compared to control mice, particularly after running tests and in trained mice. This suggests that glycolysis in muscle tissue is reduced after treatment with GW501516, and becomes more apparent immediately after the exhaustive running (Fig. 1 and Supplementary Fig. S1 online). Consistent with this reduced glucose metabolism, increased serum galactose was also only observed after GW501516 treatment. Although glycolytic metabolism of galactose yields no net adenosine triphosphate (ATP), previous research demonstrated that galactose induces a metabolic shift towards a more oxidative phenotype and enhances aerobic mitochondrial metabolism in myotubes^22^.

PPARδ is intricately involved in enhanced running endurance. It mediates oxidative adaption following exercise training and increased expression in skeletal muscle was reported after both acute exercise and endurance training^12^,^23^,^24^. Similar to chemical activation of PPARδ, endurance training increases fatty acid metabolism in skeletal muscle and reduced lactate production during exercise^25^. Accordingly, the running distance of untreated trained (TN) mice is over 100% greater of that of untreated untrained (NN), blood glucose levels in the TN group are significantly higher and blood lactate levels are significantly lower than those measured in the NN group. Enhanced fatty acid oxidation in skeletal muscle was also observed by measuring elevated components of the fatty acid oxidation pathway including palmitic acid and octanoic acid. Caproic acid and butyric acid were not significantly different between groups in our assay, but the sensitivity to detect these metabolites may be insufficient. The underlying mechanism for PPARδ and PPARα activation to increase fatty acid oxidation in skeletal muscle is by upregulating components of the electron transport chain (CYT-C and UCP3) and CPT1b in the mitochondria. Serum inositol is also increased by training and may help increase endurance in trained mice. Inositol can help transport fat from the liver and improve the distribution of fat throughout the body, allowing it to be used more efficiently as an energy source. Our results further corroborate that exercise training promotes fat mobilization and fatty acid oxidation as energy substrates during running. Furthermore, this is likely mediated through exercised-induced PPARδ expression in a way that is similar to what was reported previously in human skeletal muscle following exercise training^12^.

One of the most striking differences between drug treatment and exercise was the increase of serum PUFA and hydroxybutyrate. Compared to training, GW501516 increased levels of UFA, especially PUFA, and they were further increased by the combination of GW501516 and training. This is in agreement with previous studies that showed increases of enzymes that produce UFA after treatment with a PPARδ agonist^5^,^21^. Our study demonstrated GW501516 increased levels of 3 PUFA (α-linolenic acid, arachidonic acid, and its precursor 8,  11,  14-Eicosatrienoic acid) regardless of exercise training. Previous studies have demonstrated that all long-chain fatty acids and their derivatives are putative PPAR ligands^2^,^26^,^27^. Endogenous fatty acids that have been shown to activate PPAR include PUFAs such as some ω-3 PUFA (e.g. α-linolenic acid and docosahexaenoic acid), some ω-6 PUFA (e.g. linoleic acid and arachidonic acid), and some saturated fatty acids (e.g. stearic acid)^2^,^3^,^26^,^27^. UFA and PUFA in particular, could act as activators of PPARδ after being increased by GW501516. Additionally they are sensed by the liver which then mobilizes fatty acids and stimulates hepatic fatty acid oxidation^28^. We observed hydroxybutyrate, the major constituent of ketone bodies, was significantly increased by GW501516, but not exercise. Leucine, a ketogenic amino acid, was also increased by GW501516 only. However, exercise did increase other BCAA. Increased hepatic synthesis of ketone bodies would be expected during increased release of fatty acids from fat stores and further suggests that GW501516 promotes hepatic fatty acid oxidation that can provide muscle with an alternative energy source during exercise. Ketone bodies are an important energy substrate under stress and hydroxybutyrate can freely enter muscle mitochondria and be metabolized to acetyl CoA.

The impacts of training or GW501516 treatment on essential amino acids were slightly different. The glycogenic amino acids that can be metabolized to form pyruvate (alanine, serine and threonine) were only significantly increased after training alone. This suggests more energy is utilized from pyruvate after training. Elevations in these amino acids were reversed by GW501516 treatment suggesting that GW501516 inhibits gluconeogenesis or the conversion of glycogenic amino acids into pyruvate that was induced by training. This indirectly suggests that GW501516-treated mice specifically rely on fatty acids as energy. Three BCAA (valine, leucine, and isoleucine) were increased by both GW501516 and training. Long-distance running involves mobilization of BCAA^29^ and many studies demonstrate that BCAA concentrations immediately after exercise are reduced in the serum, but not in muscle^30^. We found that training or GW501516 treatment, preserved serum BCAA levels after exhaustive running. As essential amino acids, BCAA are primarily metabolized in muscle and are important for protein synthesis, neurotransmitter synthesis, and repair of exercise-induced skeletal muscle damage^31^,^32^. Increased BCAA may both act as an energy source (Supplementary Fig. S3 online) and protect mitochondria from exercise-induced damage to preserve mitochondrial function. In addition, increased serum BCAA may reduce tryptophan uptake in the brain and delay fatigue by competitively inhibiting 5-hydroxytryptamine (5-HT) synthesis^31^,^32^.

Metabolomic analyses are relatively new technology. It is limited by the complexity of techniques, high inter- and intra- subject variability, incomplete metabolite databases, and incomplete understanding of metabolic pathways. Although there are a total of 122 peaks measured in serum samples, only 43 peaks could be matched to known metabolites.

In conclusion,this study demonstrated that 3-week treatment with GW501516 increased running performance of both trained and untrained mice. Like training, GW501516 promoted mitochondrial fatty acid oxidation and increased fat metabolism in muscle tissue (Summarized in Supplementary Fig. S4 online). However, exercise increased energy supply by promoting catabolism of protein, glycolysis and glucogenesis from amino acids, while GW501516 increased fatty acid oxidation through BCAA and ketone body pathways.

## Materials and methods

### Chemicals and reagents

GW501516 were synthesized by the New Drug Design Center at our institute as previously reported^33^.

### Animals and treatments

Male KM mice (8 weeks of age) purchased from Vital River Laboratory Animal Technology Co. Ltd. (Beijing, China) were randomly divided into 4 groups: untrained controls (NN); trained controls (TN); untrained mice that received GW501516 treatment (NG); and trained mice that received GW501516 treatment (TG). GW501516 was dissolved in DMSO and suspended in 0.5% carboxymethyl cellulose. Mice were housed under a constant 12-h day/night schedule at a temperature of 22 °C with *ad libitum* access to standard laboratory rodent chow and water. Prior to experiments, all mice were acclimated to moderate wheel running (YIYAN Science & Technology Development Co., Ltd, SHANDONG, China) every other day (15 rpm [0.6 m/revolution] for 20 min) for 1 week. Untrained mice were then left sedentary while trained mice were subjected to training (20 rpm for 30 min/day; 5 days/week) with moderate degree of running difficulty. After 3 weeks of training and/or GW501516 treatment (5 mg/kg/day), running endurance performance was measured by a wheel running test, where the speed was set to17 rpm for the first 3 hours and then was increased by 4 revolutions per hour until exhaustion (exhaustion was defined when mice were unable to avoid 5 electrical shocks in a 15 minute period). Blood glucose and lactate concentrations were measured before and immediately after running tests using the BIOSEN C_line glucose and lactate analyser (EKF-diagnostic, Barleben, Germany). After running endurance tests, blood samples for metabolomics assays were taken from the retro-orbital sinus following euthanasia with ether. The gastrocnemius was then isolated immediately after decapitation, frozen, and stored at −80 °C.

In a separate study, mice treated with GW501516 were euthanized andgastrocnemii were dissected and immediately frozen in liquid nitrogen-cooled isopentane. SDH staining was performed on these samples as previously described (13). All animal handling and experiments were performed strictly in accordance with the recommendations of the Guide for the Care and Use of Laboratory Animals of the National Institutes of Health. The experimental protocol was approved by the Animal Experimental Ethics Committee of the Beijing Institute of Pharmacology and Toxicology.

### Metabolomic analyses

Sample preparation and metabolomic analyses were performed as previously described^34^. For fatty acid analyses, 200 μl of serum was placed into a clean tube and spiked with an internal standard (20 μl of heptadecanoic acid methyl ester). The mixed solution was treated with methanol containing 2.5% sulfuric acid and vortexedfor 30 s. After being placed on a shakerat 80 °C for 90 min and then cooled, 1.5 ml of 0.9% sodium chloride was added and samples were then centrifuged at 3000 rpm for 5 min. The supernatant was transferred into a vial dried under N_2_ at 45 °C. The residue was dissolved in 40 μl of n-hexane, sonicated for 2 min, and then centrifuged at 13000 rpm for 10 min. Then supernatant was then taken and kept at 4 °C until measurements were performed.

Aliquots of the prepared samples (1 μL) were injected at a split ratio of 1:5 into an Agilent 6890N gas chromatography (GC) unit coupled with a Pegasus HT time-of-flight mass spectrometer (MS) (Leco Corporation, St Joseph, MI). Separation was achieved on an Rsi-5MS (30 m × 250 μm (i.d.) × 0.25 μm) fused silica capillary primary column (Agilent J&W Scientific, Folsom, CA, USA) and an RTX-200 (1.590 m × 180 μm (i.d.) × 0.20 μm) fused silica capillary secondary column (Restek Corp., Belle-fonte, PA, USA) with helium as the carrier gas at a constant flow rate of 1.0 ml/min. The temperature of the injection was 260 °C, the transfer interface temperature was 280 °C, and the temperature of the ion source was 220 °C. The GC temperature program was set to 1 min of isothermal heating at 70 °C followed by a 5 °C/min increase to 280 °C that was held for 2 min. The secondary column was maintained at a temperature 10 °C greater than the primary column and the modulator temperature offset was 15 °C greater than the secondary column. The solvent acquisition delay was 180 s. Electron impact ionization (70 eV) at a detector voltage of 1,450 V in full scan mode (m/z 50–800) was used with an acquisition rate of 100 spectra/second in GC × GC-TOFMS mode. A modulation period of 6 s with a hot pulse time of 1.2 s was used.

Chromatography acquisition, baseline correction, noise reduction, smoothing, peak area calculations, and references to spectral databases were performed using the ChromaTOF software (Version 4.5, Leco Corp.). Peaks with signal-to-noise (S/N) ratios over 100 were analysed and peak areas were calculated by the software using unique mass. Peaks with a similarity index (SI) more than 60% were assigned the compound identities and verified with the available reference compounds. Peaks with less than a 60% SI were considered as unknown compounds. Alignment, normalization, and determination of Fisher ratios were performed with the Statistical Compare feature in ChromaTOF software.

### *q*PCR

To determine the relative mRNA expression levels of PPARδ-related genes, total RNA was isolated from gastrocnemius samples and real-time PCR with the ABI PRISM 7300 sequence detection system (Applied Bio systems) was performed as previously described^35^. Primers used in the qPCR are as previously reported^36^. The relative amounts of each mRNA were calculated using the comparative CT method. mRNA levels are expressed as fold changes relative to expression of β-actin.

### Data analyses

All results are expressed as mean ± SEM. Data sets were analysed and validated by univariate and multivariate statistical methods. After peak alignment and normalization, data sets were imported into SIMCA-P 12.0 software (Umetrics, Umeå, Sweden). PCA and OPLS-DA were performed to visualize changes in metabolic profiles between groups after mean centering and Pareto scaling. A default seven-fold internal cross validation was used to extract R^2^ values that represent the explained variance and Q^2^ values indicating predictive capability. Scatter plots of scores were used to visualize the variability between samples, while loading plots with confidence intervals, sorted according to VIP values, highlighted the main variables (compounds) accounting for differences between groups. The metabolites that were significantly different between groups (p < 0.05) were identified from the loading plots of the OPLS-DA.

Statistical analyses were performed by two-tailed Welch’s t-tests, one-way analysis of variance (ANOVA) followed by Tukey’s multiple comparison tests, or 2-way ANOVAplus repeated measurements.SPSS 19.0 software was used for all statistical calculations (SPSS Inc., Chicago, IL, USA). *P* values < 0.05 were considered statistically significant. Both multivariate and univariate approaches were used to verify the significance of each metabolite in the GW501516 treatment group from the controls. Metabolic pathway analyses and identification of possible biological roles were done using MetaboAnalyst (http://www.metaboanalyst.ca/) based on the KEGG (http://www.genome.jp/kegg/).

## Author Contributions

Conceived and designed the experiments: W.C. and L.L.W. Performed the experiments: W.C., R.G., X.N.X. and H.J.L. Analyzed the data: W.C., R.G., F.T.D. and L.L.W. Contributed reagents/materials/analysis tools: Z.B.Z., F.T.D. Scientific discussion and guidance: W.C., R.G., S.L., F.T.D. and L.L.W. Wrote the paper: W.C., R.G., F.T.D. and L.L.W. All authors have read and approved the manuscript for publication.

## Additional Information

**How to cite this article**: Chen, W. *et al*. A metabolomic study of the PPARd agonist GW501516 for enhancing running endurance in Kunming mice. *Sci. Rep.* 5, 09884; doi: 10.1038/srep09884 (2015).

## Supplementary Material

Supplementary Information
