# Supplementary material for: A metabolomic study of the PPARδ agonist GW501516 for enhancing running endurance in Kunming mice
Source: Sci Rep. 2015 May 6;5:9884. doi: 10.1038/srep09884 (PMC4421799; doi:10.1038/srep09884)
Supplement: Supplementary Information [file srep09884-s1.pdf]

# A metabolomic study of the PPARδ agonist GW501516 for enhancing running endurance in Kunming mice

Wei Chen[1,*], Rong Gao[2*], Xinni Xie, Zhibing Zheng[1], Haijing Li[2], Song Li[1], Fangting Dong[2, #], Lili Wang[1, #]

1.Beijing Institute of Pharmacology and Toxicology, Beijing 100850, China

2.National Center of Biomedical Analysis, Beijing 100039, China

* Wei Chen and Rong Gao have contributed equally to this work.

# Author for correspondence:

Fang-Ting DONG

National Center of Biomedical Analysis, Beijing 100039, China

Fax +86-10-68186281

E-mail: dft@proteomics.cn

Lili Wang, Ph.D.

Beijing Institute of Pharmacology and Toxicology, Beijing, China

Fax: 86-10-6824-0321

E-mail: wangll63@126.com

**Supplementary Figure 1**

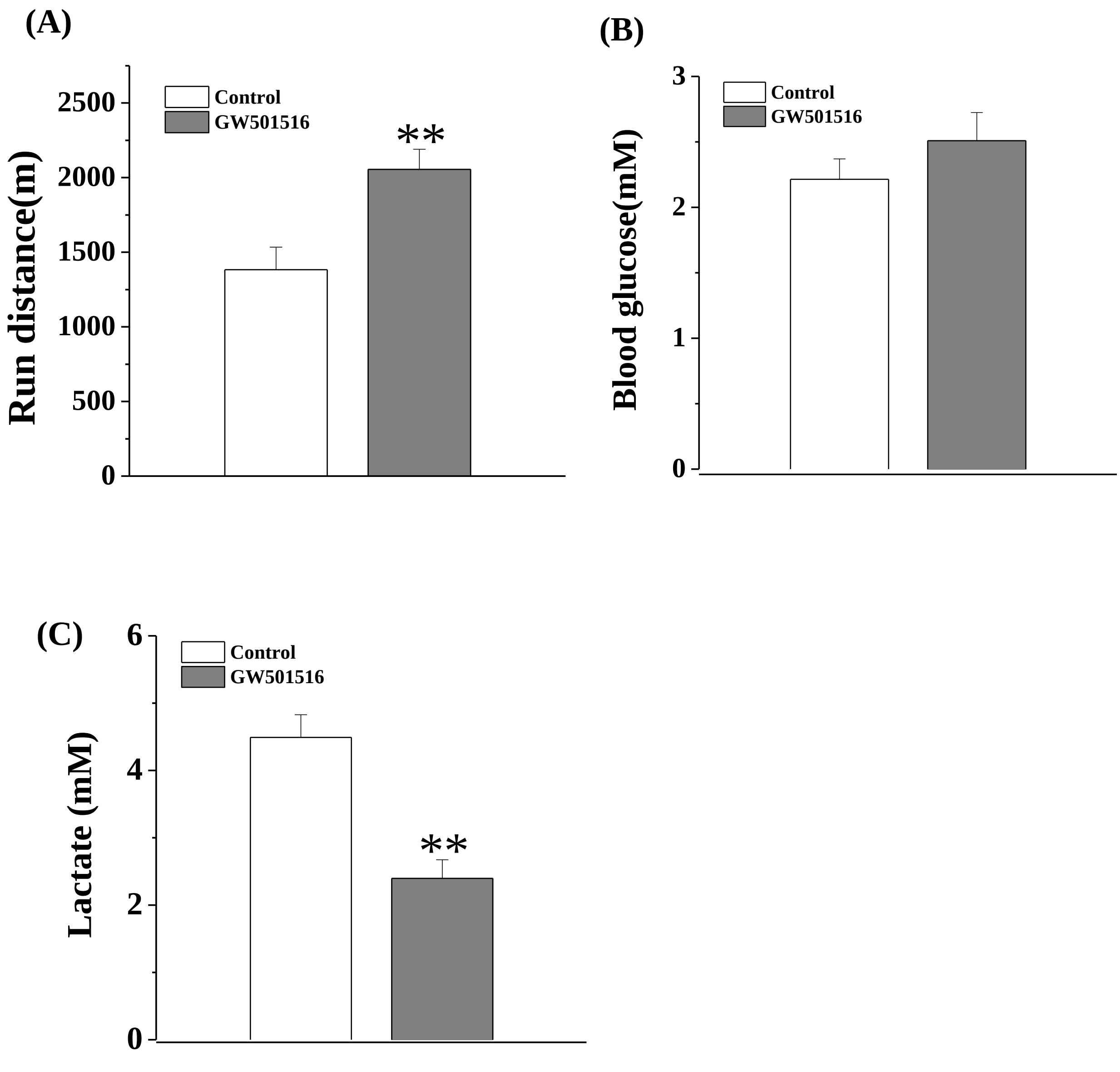


**Figure S1. Effects of GW501516 administration for 7 days on running performance in sedentary mice.** Forced treadmill running endurance tests were performed after 7 days of GW501516 treatment. Blood glucose and lactate were tested immediately after running (n = 9–10 per group). *p<0.05, **p<0.01 compared to untreated control mice.

**Supplementary Figure 2**

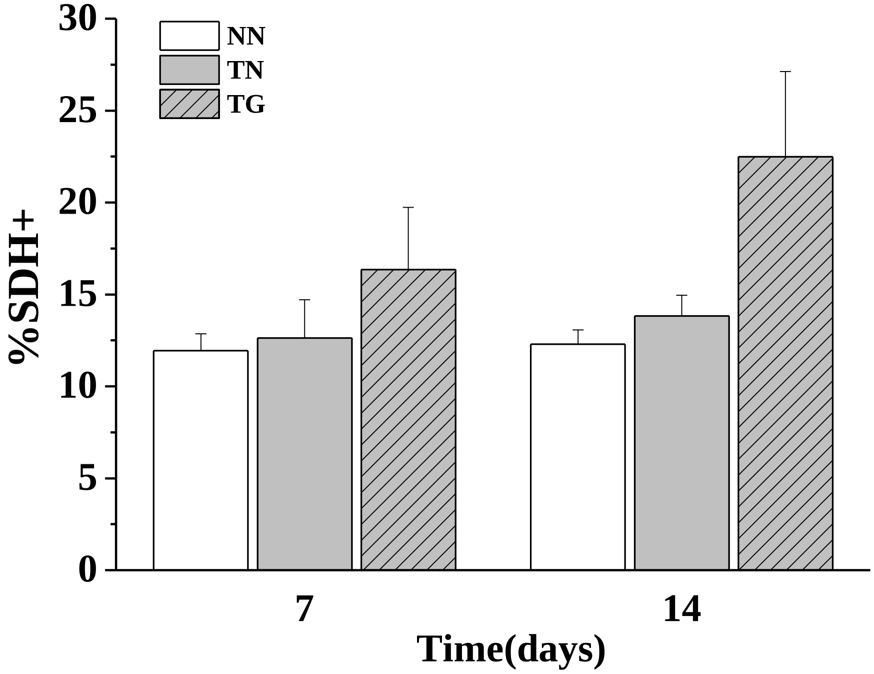


**Figure S2. Effects of GW501516 treatment on SDH-positive fibres in trained mice.**

Quantification of SDH-positive fibres from the NN, TN, and TG groups after 1 and 2 week of treatment.

**Supplementary Figure 3**

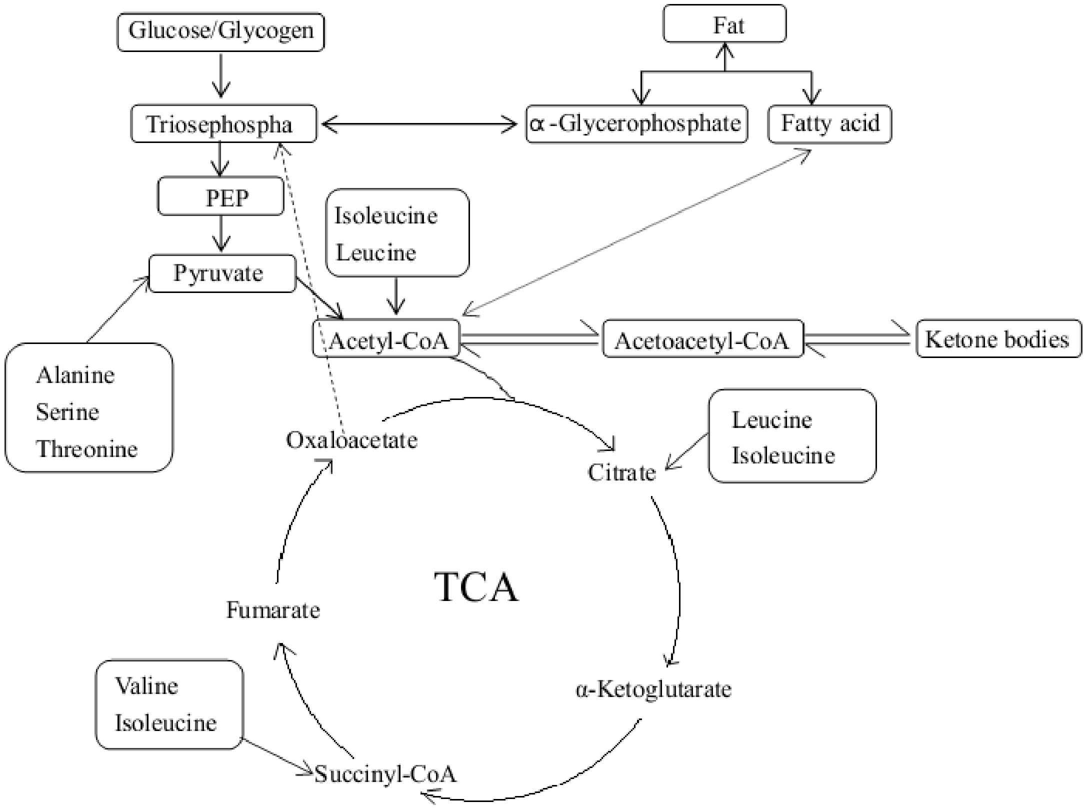


**Figure S3. Diagram of branched-chain amino acids (BCCA) in the tricarboxylic acid cycle.**

**Supplementary Figure 4**

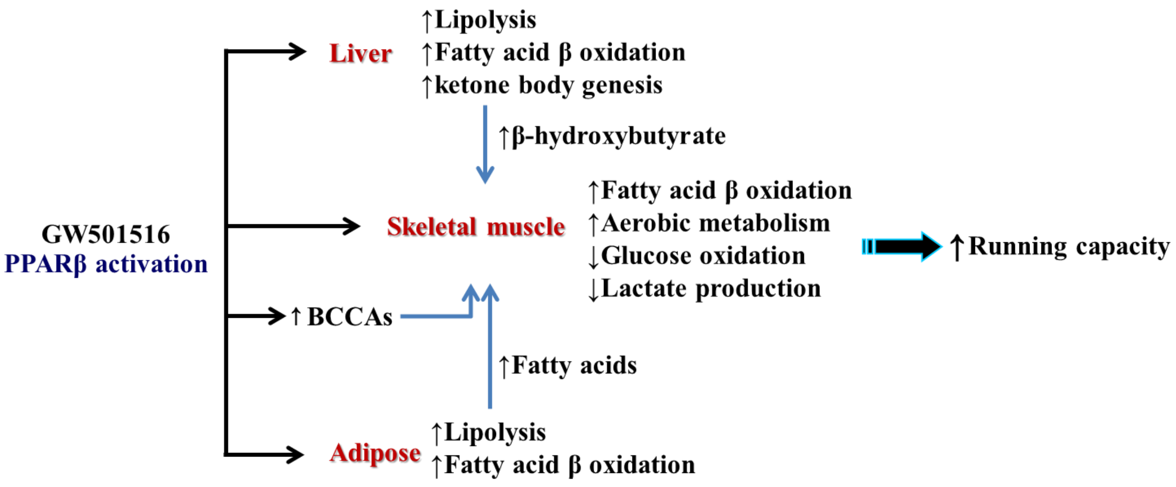


**Figure S4. Schematic of the physiological pathways that enhance running endurance after GW501516 treatment.** PPAR δ is activated by GW501516 leading to reduced blood glucose metabolism and increased fatty acid metabolism by promoting lipolysis in adipose tissue and the liver, which increases the supply of fatty acids, β-hydroxybutyrate, and BCCAs to skeletal muscle.

**Supplement Table 1**

**Summary of the differentiated serum fatty acids associated with training and/or GW501516 treatment**

| NO. | Metabolites | *P* | | | | VIP | | | |
|---|---|---|---|---|---|---|---|---|---|
| | | **a** | **b** | **c** | **d** | **a** | **b** | **c** | **d** |
| 1 | 3-hydroxy-Hexadecanoic acid | 0.001 | 0.002 | 0.373 | 0.002 | 1.48 | 1.84 | 0.77 | 1.36 |
| 2 | Octanoic acid | 0.512 | 0.001 | 0.704 | 0.003 | 0.34 | 1.38 | 0.41 | 1.17 |
| 3 | Decanoic acid | 0.200 | 0.010 | 0.485 | 0.003 | 0.65 | 1.13 | 0.81 | 1.28 |
| 4 | Dodecanoic acid | 0.000 | 0.043 | 0.608 | 0.004 | 1.69 | 0.89 | 0.07 | 1.32 |
| 5 | Tetradecanoic acid | 0.000 | 0.000 | 0.050 | 0.003 | 1.68 | 1.48 | 1.12 | 1.35 |
| 6 | Palmitic acid | 0.109 | 0.013 | 0.003 | 0.000 | 0.82 | 1.19 | 1.82 | 1.37 |
| 7 | 15-methyl-Hexadecanoic acid | 0.000 | 0.217 | 0.720 | 0.001 | 1.91 | 0.74 | 0.40 | 1.40 |
| 8 | Pentadecanoic acid | 0.000 | 0.032 | 0.197 | 0.001 | 1.89 | 0.97 | 0.66 | 1.48 |
| 9 | Stearic acid | 0.000 | 0.005 | 0.003 | 0.000 | 1.18 | 0.00 | 1.84 | 1.38 |
| 10 | Eicosanoic acid | 0.006 | 0.377 | 0.829 | 0.005 | 1.34 | 0.28 | 0.07 | 1.29 |
| 11 | Docosanoic acid | 0.002 | 0.351 | 0.756 | 0.016 | 1.45 | 0.20 | 0.11 | 1.11 |
| 12 | Tetracosanoic acid | 0.001 | 0.874 | 0.416 | 0.007 | 1.60 | 0.30 | 0.30 | 1.24 |

| | | | | | | | | | |
|---|---|---|---|---|---|---|---|---|---|
| 13 | Palmitoleic acid | 0.031 | 0.924 | 0.445 | 0.045 | 1.03 | 0.05 | 0.65 | 1.18 |
| 14 | Oleic acid | 0.839 | 0.839 | 0.018 | 0.046 | 0.10 | 0.58 | 1.64 | 1.19 |
| 15 | Linoleic acid | 0.008 | 0.533 | 0.358 | 0.046 | 1.30 | 0.36 | 0.62 | 0.92 |
| 16 | α-linolenic acid | 0.000 | 0.417 | 0.000 | 0.263 | 1.78 | 0.17 | 2.24 | 0.51 |
| 17 | 11,14-Eicosadienoic acid | 0.628 | 0.397 | 0.002 | 0.004 | 0.25 | 0.64 | 1.94 | 1.36 |
| 18 | 8,11,14-Eicosatrienoic acid | 0.000 | 0.027 | 0.043 | 0.422 | 1.63 | 0.94 | 1.49 | 0.19 |
| 19 | Arachidonic acid | 0.003 | 0.112 | 0.004 | 0.022 | 1.40 | 0.63 | 1.83 | 1.06 |
| 20 | 8,11,14,17-Eicosapentaenoic acid | 0.179 | 0.506 | 0.011 | 0.042 | 0.69 | 0.38 | 1.13 | 1.02 |
| 21 | Eicosapentaenoic acid | 0.005 | 0.036 | 0.433 | 0.020 | 1.42 | 1.03 | 0.66 | 1.12 |
| 22 | Docosahexaenoic acid | 0.906 | 0.673 | 0.037 | 0.010 | 0.06 | 0.02 | 1.54 | 1.21 |

1. *P* means *p* value obtained from one-way analysis of variance (ANOVA).
2. Variable importance in the projection (VIP) was obtained from PLS-DA.
3. a, b, c, d respectively represents NG vs. NN, TN vs. NN, TG vs. TN, TG vs. NG.
